# Awareness, knowledge, and behaviors regarding trans unsaturated fatty acids in a sample of Lebanese adults

**DOI:** 10.1002/fsn3.4211

**Published:** 2024-05-19

**Authors:** Marianne El Hajj, Jennifer Abou Chaaya, Jessica Abou Chaaya, Maya Tueni

**Affiliations:** ^1^ Department of Nutrition and Dietetics, Faculty of Public Health Lebanese University Fanar Lebanon; ^2^ Department on Internal Medicine, Faculty of Medicine American University of Beirut Medical Centre Beirut Lebanon

**Keywords:** awareness, behaviors, knowledge, Lebanon, Middle East, partially hydrogenated oils, trans fatty acids

## Abstract

Lebanon is a country in the Middle East that had been witnessing nutrition transition to a westernized diet high in trans fats (TFs) and saturated fatty acids (TFAs) that had been linked to cardiovascular diseases and many other health issues. This study examines TF‐related awareness, knowledge, and self‐reported behaviors among a sample of Lebanese adults aged between 18 and 64 years, as well as their association with sociodemographic factors and anthropometric measurements. Using a multicomponent questionnaire, a cross‐sectional study was conducted online, across all Lebanese regions based on a convenience sampling method (*n* = 401). Factors associated with TF‐related awareness, knowledge, and behaviors were examined by multivariate linear regression analysis. The study highlighted specific gaps in TF‐related awareness, knowledge, and behavioral practices as well as differences by sociodemographic factors. Most of the participants (36%) had heard of partly hydrogenated oils (PHOs) rather than TFs (49%). A higher proportion of respondents (54%) said they understood a little about TFs, the majority had inadequate knowledge about the foods that contain TFs, and 44% said they would not give up eating their favorite snack even if they knew it contains TFs. Overall, consumers' awareness and knowledge about TFs are rather low and the majority had fair behavioral practices. In addition, being a woman and having higher education level were significantly associated with higher levels of TFs awareness, knowledge, and behavior scores. Higher behavior scores were shown in older participants, married, and those who had part‐time jobs, whereas having higher income and normal weight were significantly associated with higher awareness scores. These findings offer valuable insight into TF‐related awareness, knowledge, and behaviors in a sample of Lebanese adults and provide key information that could spur the development of evidence‐based TFs reduction interventions specific to the Middle East.

## Graphical abstract

Trans fats are currently widely consumed in our population and leading to hazardous health diseases. Lebanon, a third world country, is among the highest consumers of trans fats and has a high prevalence of cardiovascular disease. Our study revealed the lack of knowledge, awareness, and behaviors of the Lebanese population regarding trans fats.

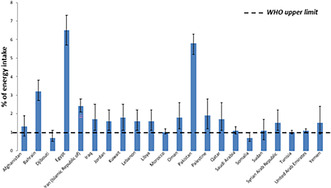

## 1 INTRODUCTION

Trans fats (TFs), also referred to as trans fatty acids (TFAs), are types of unsaturated fatty acids (UFAs) that occur naturally as well as synthetically. Natural TFs are derived from ruminants as a byproduct of anaerobic bacterial fermentation. Consequently, animal fats, such as those found in meat and dairy, contribute to the presence of TFAs in the diet (Brouwer et al., 2010; Lichtenstein, 2016). Conversely, industrially produced TFs are a result of the hydrogenation process of vegetable oils, leading to the formation of “partially hydrogenated” oils (PHOs) (Brouwer et al., 2010). The introduction of PHOs into the food supply occurred in the late nineteenth and early twentieth centuries, marking the emergence of industrially produced trans fatty acids (IP‐TFAs). Initially, IP‐TFAs served as a cost‐effective substitute for animal fats and were utilized to prolong the shelf life of various oils and foods by reducing their susceptibility to oxidation, as opposed to polyunsaturated fatty acids which are prone to autoxidation (World Health Organization [WHO], 2019). Common sources of these oils include baked and fried foods, prepackaged snacks, cooking oils, and spreads. The popularity of TFs increased following the discovery of the adverse health effects associated with saturated fatty acids (SFAs) during the 1950s and 1970s (WHO, 2019).

**FIGURE 1:**
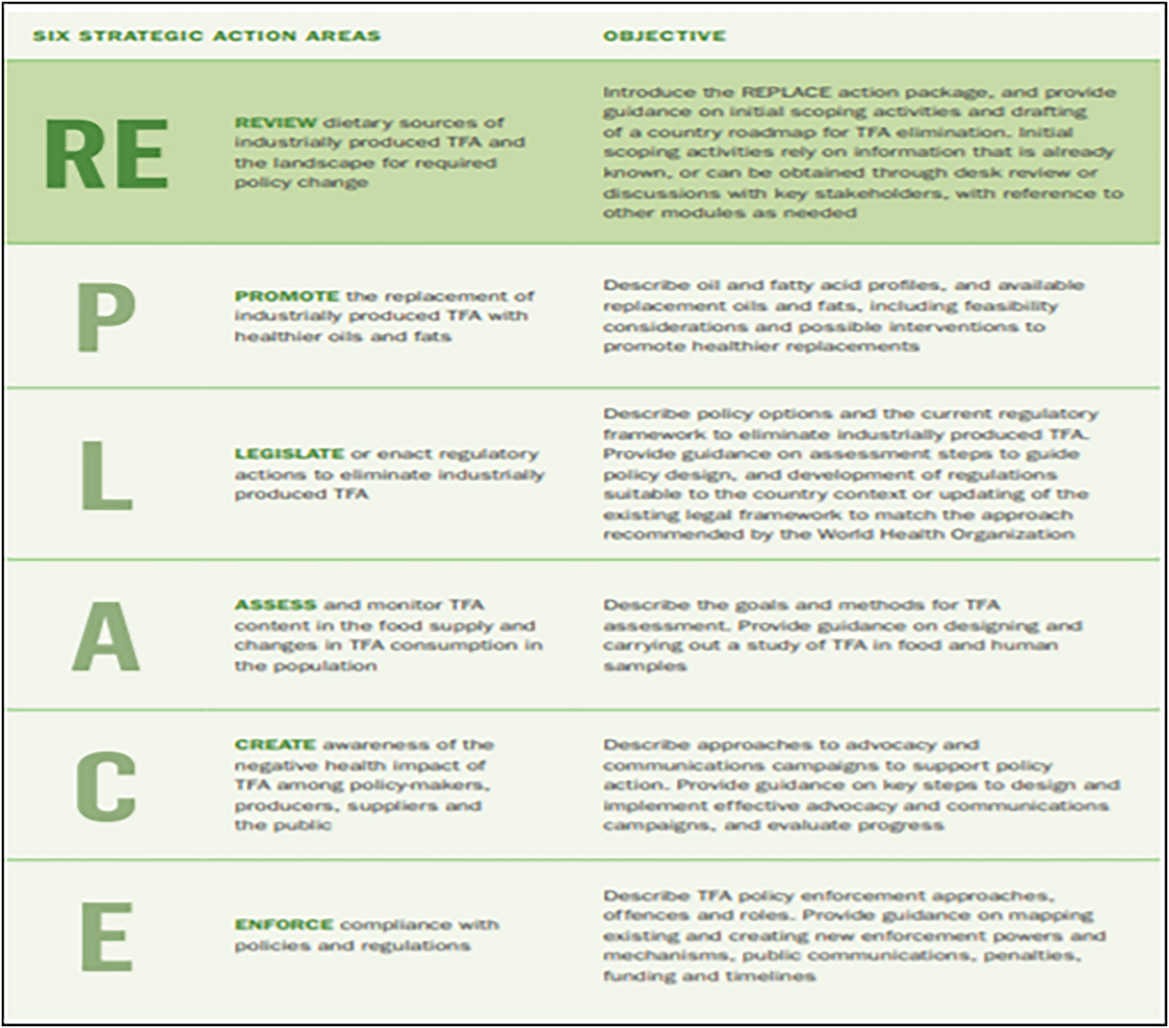
Modules of the REPLACE action package (WHO, 2019c).

However, by the late twentieth century, a large body of evidence revealed TFAs' negative metabolic effects and the link between TFAs intake and cardiovascular diseases (CVDs), particularly coronary heart disease (CHD) (de Souza et al., 2015; Guasch‐Ferré et al., 2015; Wang et al., 2016). In fact, TFs rich diets increase the risk of heart disease by 21% and death by 28% (WHO, 2018a). Since then, reducing/eliminating TFs has been one of the most important national and worldwide objectives (Harvard T.H. Chan School of Public Health, 2018; WHO, 2018b). Several initiatives have started to achieve this goal such as the World Health Organization (WHO). WHO recommends that total TFs intake be kept to <1% of total caloric intake (TCI), or <2.2 g/day on a 2000‐calorie diet (WHO, 2018c). In May 2018, the REPLACE action (Figure 1) package was launched to provide strategic direction to all countries in achieving elimination of IP‐TFAs from the global food supply by 2023. This was achieved by guiding governments to change public policies, improving regulations, and food manufacturing practices, as well as providing TF replacement options (WHO, 2018e). In fact, replacing TFs with UFAs like polyunsaturated fatty acids (PUFAs) decreases the risk of heart disease, in part, by ameliorating the negative effects of TFs on blood lipids (WHO, 2019a). High‐income countries (HIC) were among the first responders, whereas more action is needed in low‐ and middle‐income countries (LMIC), where IP‐TFAs controls are often weaker (WHO, 2018b), especially as these countries experience a nutrition transition, with a shift toward unhealthy diets and sedentary lifestyles (WHO, 2019b). Lebanon is one of these countries witnessing transition with a trend toward eating more processed foods and foods rich in TFs especially among younger ages (Farhat et al., 2016). CVD is the leading cause of death in the country accounting for 47% of all‐cause mortality according to the WHO (WHO, 2018d). A similar transition is noted in the Eastern Mediterranean Region (EMR) where the rates of CVDs are among the highest in the world and contribute to more than two in five deaths (WHO, 2019b).

Educational programs about TFs represent one strategy employed at the individual level to empower the public to make healthier choices in their daily lives (Eckel et al., 2009; Pletzke et al., 2010). Such initiatives have demonstrated positive effects on consumers' understanding of TFs and their purchasing habits. The knowledge–attitude–behavior (KAB) model is commonly utilized to guide nutrition education efforts (Pletzke et al., 2010). This model operates on the premise that knowledge is pivotal in influencing behavioral changes and that individuals can acquire knowledge and skills through education (Liu et al., 2016). Consequently, it is imperative to assess public awareness, knowledge, and behaviors to effectively deliver educational interventions. Research conducted in several developed nations has uncovered significant gaps in public knowledge concerning TFs, their dietary sources, and the specific measures necessary to reduce their consumption (Jasti & Kovacs, 2010; Nasser et al., 2011). However, most of these studies have been conducted in high‐income countries and Western nations (Ellis & Glanville, 2010; Jasti & Kovacs, 2010; Nasser et al., 2011), limiting their generalizability to LMICs.

**FIGURE 2:**
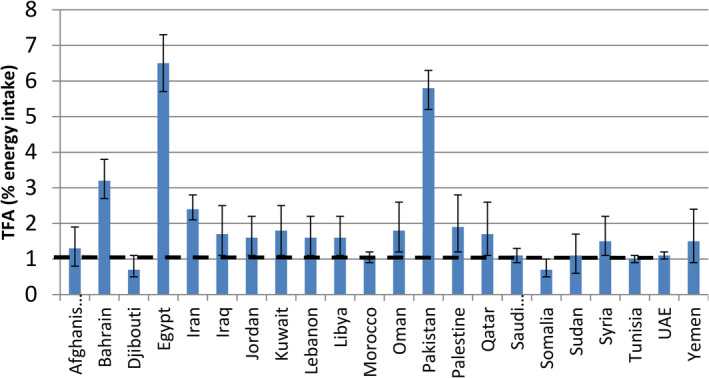
Trans fats intakes in countries of the region (trans fatty acids as percentage of energy intake) (Jawaldeh & Al‐Jawaldeh, 2018). WHO, World Health Organization.

While scant literature addresses the issue of TFs in the Arab world and Eastern Mediterranean Region (EMR), some reports have highlighted TFs consumption in certain countries. Estimates vary, ranging from 0.28% of energy intake (EI) in Tunisia (Jawaldeh & Al‐Jawaldeh, 2018) to 6.5% EI in Egypt (Micha et al., 2014). In Lebanon, a recent study spanning from 2019 to 2021 found that 93% of tested products met the WHO recommendations of less than 1% of total caloric intake (TCI), while approximately 7% exceeded this threshold (Hoteit et al., 2021a, 2021b). However, TFAs intake in Lebanon was reported to be 2.3% of EI in 2016 (Farhat et al., 2016), surpassing the WHO guidelines (WHO, 2018c) (Figure 2).

As far as we are aware, there is a dearth of research on awareness, knowledge, and behaviors related to TFs in Lebanon. Consequently, the level of awareness, knowledge, and behaviors regarding this type of fat among the Lebanese population remains unclear. Therefore, the objectives of our study are twofold: first, to assess awareness, knowledge, and self‐reported behaviors regarding TFs among a sample of Lebanese adults aged 18–64 years. Second, to explore the relationship between awareness, knowledge, and behaviors related to TFs and various sociodemographic factors and anthropometric measurements.

## 2 MATERIALS AND METHODS

### 2.1 Study design and subjects

A cross‐sectional survey was conducted among Lebanese adults aged between 18 and 64 years from all over Lebanon based on the six governorates: Beirut, Mount Lebanon, North, Beqaa, South, and Nabatiyeh. It was conducted from March to August 2021. Convenience sampling method was adopted. Participants were selected by sending an online invitation link of the survey developed using “Google Forms” through different platforms on social media as most of the population use these platforms in their daily lives. Sample size was calculated based on the following formula (Cochran), with a confidence interval (CI) of 95%:

$$N=z^{2}\times P\times \left(1-P\right)/e^{2}$$

*z* = level of confidence (for a level of confidence of 95%, *z* = 1.96); *P* = estimated proportion of the population that presents the characteristic *P* = .5 (since previous studies have not provided sufficient information about TFs awareness, an assumption was made that half of the subjects are familiar with TFs); *e* = margin of error, *e* = 0.05; *N* (sample size) = 384 participants.

Electronic consent was obtained from all participants before starting the online questionnaire. All consenting participants were volunteers and free to withdraw at any time (Appendix S1). The subject's privacy and the confidentiality of the collected data were maintained.

### 2.2 Inclusion and exclusion criteria

Total of 413 responses were collected, from which 9 duplicates were identified and removed due to repeated entries. Additionally, 3 responses were excluded due to missing values, resulting in a final sample size of 401 responses.

### 2.3 Study instruments and data collection

The study encompassed the development of a multicomponent questionnaire consisting of six parts: sociodemographic information, anthropometric measurements, general health inquiries, TFs awareness, TFs knowledge, and TFs behaviors (Appendix S2).

The first part inquired about socioeconomic and ‐demographic information. This section included seven questions inquiring about the gender, age, place of residence, education level, total income, employment status, and marital status. The age and the place of residence were recategorized for analysis purposes. Age groups were divided into three categories (18–34, 35–54, and 55–64 years). On the other hand, the place of residence was regrouped into four areas: Beirut and Mount‐Lebanon, South and Nabatieh, North, South, and Bekaa. Concerning the total income per month, this was categorized into four sections according to the minimum wage during that period in Lebanon (very low: less than 750,000 Lebanese pounds [LBP]/month; low: 750,000–2,000,000 LBP/month; moderate: 2,000,000–4,000,000 LBP/month; and high: more than 4,000,000 LBP/month).

The second part of the survey collected anthropometric data, including weight, height, and calculated body mass index (BMI). The third section focused on gathering general health information, encompassing questions regarding self‐reported health status and the presence of any chronic health conditions. Self‐reported health status was categorized into four groups: excellent, very good, good, and fair/poor. Specific chronic health problems were identified based on whether respondents reported having any such conditions or not. The remainder of the questionnaire was designed to assess consumers' awareness, knowledge, and behaviors regarding TFs. As there was no existing questionnaire available, a customized questionnaire and scoring system were developed specifically for this study. The survey questions were structured and drew inspiration from previous studies for reference (Eckel et al., 2009; Ellis & Glanville, 2010; Lin & Yen, 2010; Nasser et al., 2011; Pletzke et al., 2010). These questions were closed ended to enhance response rates and minimize bias. The questionnaire was primarily written in English and then translated into Arabic, the native language of the country. Prior to implementation in the fieldwork, the questionnaire underwent a pretest phase involving a sample of 15 Lebanese adults. The following is a brief overview of the various components of the questionnaire.

#### 2.3.1 Awareness

To assess awareness of TFs, we employed seven questions, three of which centered on general nutrition topics. These questions aimed to gauge participants' ability to identify different types of fats and oils, factors influencing their food choices, and their use of food labels while grocery shopping. Additionally, four questions were specifically tailored to TFs, focusing on whether participants had heard of TFs prior to the survey and the sources of this information, their level of concern about TFs, and their associations when thinking about TFs.

#### 2.3.2 Knowledge

The knowledge section of the questionnaire investigated the relationship between TFs and participants' overall health status. They were asked about their perceived comprehension of TFs concerning health, their viewpoints on this type of fat, and their stance on the importance of TFs disclosure on food labels. Furthermore, participants were asked to recognize foods containing TFs from a list provided.

#### 2.3.3 Behaviors

One question addressed the general use of food labels, while three questions focused specifically on TF‐related labels. Additionally, five questions aimed to determine participants' endeavors to lower TFs consumption, their readiness to forego their favorite snack if it contained TFs, and any substitutions made in their food choices to decrease TFs intake. Moreover, participants were asked about the frequency of consuming fried and baked foods. Further inquiries were made regarding reducing the intake of high‐fat foods, the frequency of using various types of fats in meal preparation, and participants' behaviors when dining out at restaurants.

Response options varied from multiple and single choice questions to Likert scales. Closed questions were also used and an “I don't know” option was added to some of the questions. Each questionnaire lasts 10–15 min approximately.

### 2.4 Statistical analysis

Data were analyzed using SPSS software, version 25.0 (SPSS, Chicago, IL, USA). Descriptive statistics were performed for demographic variables as well as responses to all questions included in the questionnaire. Continuous and categorical data were expressed as mean (± standard deviation) or counts and percentages, respectively. For everyone, an awareness score was calculated based on the number of correct answers to awareness questions, with scores ranging from 1 to 28 (the higher the score, the higher the awareness). Similarly, a knowledge score was created, with scores ranging between 0 and 32 (the higher the score, the higher the knowledge toward TFs). A behavior score was created based on the number of favorable behaviors statements, with scores ranging between 9 and 54 (the higher the score, the more favorable are the behaviors toward TFs). The scores in awareness, knowledge, and behaviors practice domains were categorized as poor (less than and equal to 50%), fair (51%–69%), and good (70% and above). Thus, awareness, knowledge, and behavior scores were classified as poor (scores less than or equal to 14 for awareness, to 16 for knowledge, and to 27 for behaviors), fair (scores from 14.3 to 19.3 for awareness, from 16.3 to 22 for knowledge, and 27.5–37.3 for behaviors), and good (scores of 19.6 and plus for awareness, 22 and plus for knowledge, and 37.8 and plus for behaviors). Bivariate analyses between continuous scores and demographic, anthropometrics (BMI), and general health variables were done with independent samples *t* test, ANOVA a nonparametric test (Mann–Whitney and Kruskal–Wallis), as applicable. Pearson's chi‐square test was used to determine the association between gender and categories of awareness, knowledge, and behaviors.

Multiple linear regression modeling was conducted to identify the relationship between the dependent variables: TF awareness, knowledge, and behavior scores, and independent variables: sociodemographic factors. For each of these three dependent variables, the same procedure was used as described next. All independent variables with significant bivariate crude associations with the dependent variable were entered into a full model (all tests with *p* < .2 were considered statistically significant). Unstandardized β, the 95% CI, and *p* values were reported. The level of statistical significance for all analyses was set at *p* < .05.

## 3 RESULTS

**FIGURE 3:**
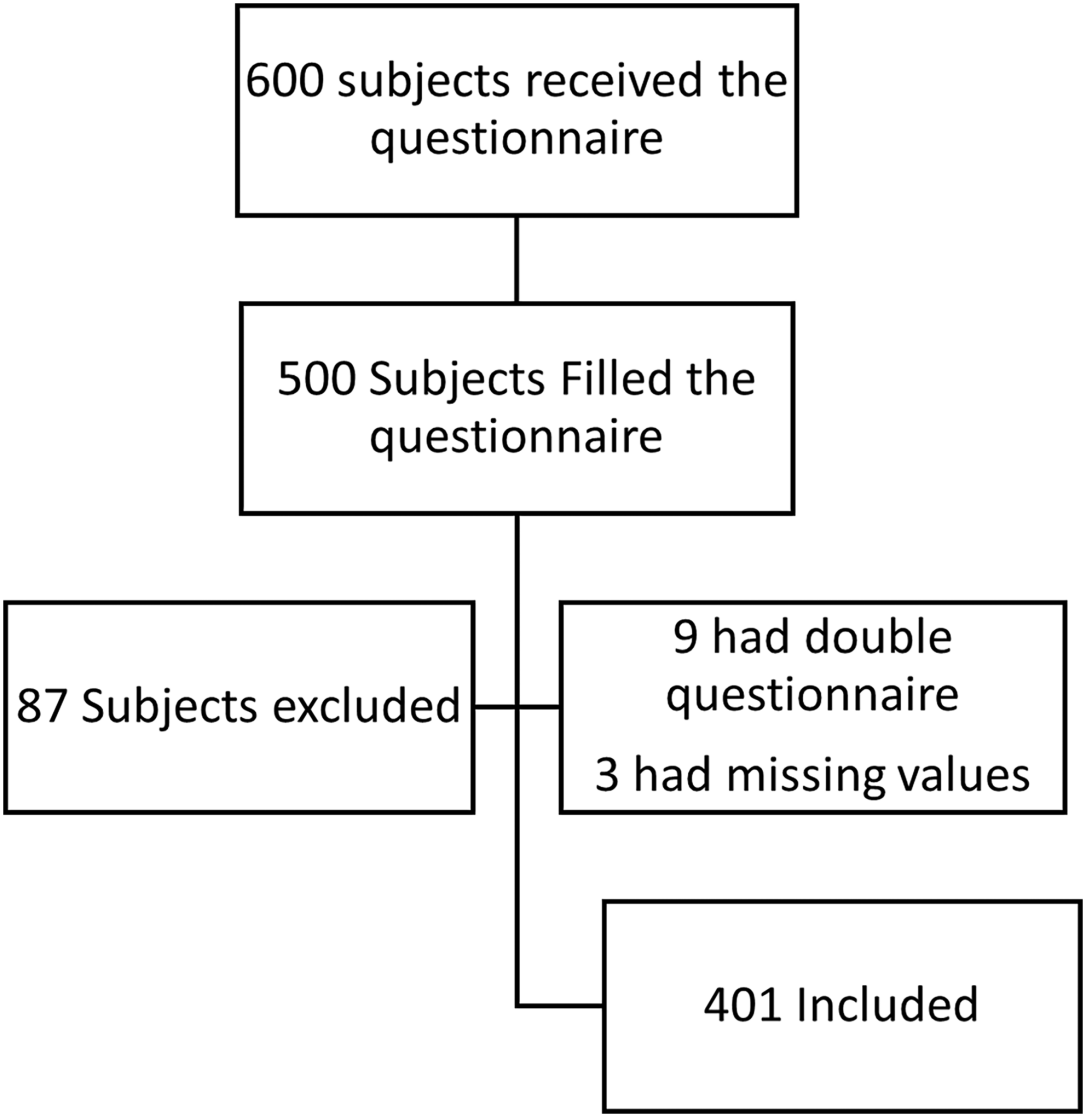
Flowchart with the number of participants included and excluded during this study.

Among the 600 questionnaire recipients, 413 responses were received. Subsequently, 9 duplicate responses and 3 with missing values were removed, resulting in a sample size of 401 responses. Additionally, 87 subjects were excluded due to not meeting the specified age criteria for the study. Response rate calculated as number of patients who responded to the survey divided by total number of questionnaires sent = (491/600) × 100 = 82% (Figure 3).

### 3.1 Sample characteristics

A total of 401 adults participated in this study. Participants' ages ranged between 18 and 64 years, with the majority (72%) falling between the ages of 18 and 34 and 68% of subjects were women. Mostly, participants were single (72%) and approximately half of them were unemployed (49%). Overall, 80% had higher education and 57% had a monthly income of less than 1,999,000 LBP/month. Of participants, 48% rated their health as very good, 70% had no health problem, and half of the participants (51%) were of normal weight (Table 1).

**TABLE 1:** Sociodemographics characteristics and general health problems of study participants (*n* = 401).

Characteristics	(*n*) 100%
Age (years)	
18–34	(288) 71.8
35–54	(84) 20.9
55–64	(29) 7.2
Gender	
Male	(130) 32.4
Female	(271) 67.6
Marital status	
Single	(290) 72.3
Married/widowed/divorced	(111) 27.7
Employment status	
Full time	(153) 28.2
Part time	(50) 12.5
Unemployed	(198) 49.4
Educational level	
Up to elementary school	(11) 2.7
High school	(69) 17.2
Undergraduate degree	(197) 49.1
Graduate degree	(124) 30.9
Total income	
Very low	(95) 23.7
Low	(132) 32.9
Moderate	(114) 28.4
High	(60) 15.0
Reported state of health	
Excellent	(53) 13.2
Very good	(140) 34.9
Good	(153) 38.2
Fair/poor	(55) 13.7
Weight status	
Underweight	(35) 8.7
Normal weight	(206) 51.2
Overweight	(100) 24.9
Obese	(60) 15
Health problem	
No	(280) 69.8
Yes	(121) 30.2

*Note*: Age in years, total income by LBP/month, weight status by BMI. As per WHO: underweight (BMI < 18.5 kg/m^2^); normal weight (18.5 ≤ BMI ≤ 24.9 kg/m^2^); overweight (25.0 ≤ BMI ≤ 29.9 kg/m^2^); obese (BMI ≥30 kg/m^2^).

Abbreviations: BMI, body mass index in kg/m^2^; LBP, Lebanese pound.

### 3.2 TF‐related awareness, knowledge, behavior scores and sociodemographic factors

TFs awareness, knowledge, and behavior scores among participants are presented in Table 2 (shown separately in Appendix Tables S1–S3). Overall, at least half of consumers had rather poor awareness (*M* ± *SD* = 14.7 ± 0.25; minimum 1, maximum 28) and knowledge scores (*M* ± *SD* = 14.4 ± 0.3; minimum 0, maximum 32), but fair behavior scores (*M* ± *SD* = 31 ± 0.26; minimum 9, maximum 54). Females outperformed males' awareness (*p* < .001), knowledge (*p* < .001), and behavior scores (*p* = .027). People aged between 18 and 34 years were significantly more likely to have higher awareness (*p* = .016) and less favorable behavior scores (*p* = .001) in comparison with older participants. Subjects who were married, widowed, or divorced, as well as those who worked part time, had higher behavior scores than singles (*p* = .014), unemployed and full‐time workers (*p* = .025). Participants with an undergraduate degree or higher education had a significantly higher awareness (*p* < .001), knowledge (*p* = .01), and behavior scores (*p* = .023) than those with a high school or less. Moreover, people with higher total income had significantly better awareness scores (*p* = .022). The analysis also revealed that people of normal weight had higher awareness scores than those who were overweight or obese (*p* = .007) (Table 2).

**TABLE 2:** Trans fat‐related awareness, knowledge, and behavior scores among a sample of Lebanese adults and their sociodemographic characteristics (*n* = 401).

Characteristics	(*n*) 100%	Awareness score (out of 28), mean ± *SD*	Knowledge score (out of 32), mean ± *SD*	Behavior score (out of 54), mean ± *SD*
Total population	(401) 100	14.7 ± 0.25	14.4 ± 0.3	31 ± 0.26
Median score [min–max]		15 [1–28]	15 [0–32]	31 [9–54]
Types of scores		Poor^a^	Poor^b^	Fair^c^
Gender				
Male	(130) 32.4	13.5 ± 4.87	12.6 ± 6.49	30.18 ± 5.26
Female	(271) 67.6	15.28 ± 5.06	15.2 ± 6.05	31.44 ± 5.31
*p*	<.001*	<.001*	.027*	
Age (years)				
18–34	(288) 71.8	15.16 ± 5.24	14.37 ± 6.61	30.4 ± 5.35
35–54	(84) 20.9	13.45 ± 4.54	14.02 ± 5.54	32.48 ± 5.08
55–64	(29) 7.2	13.86 ± 4.03	15.24 ± 5.21	33.03 ± 4.53
*p*	.016**	.67	.001**	
Marital status				
Single	(290) 72.3	14.96 ± 5.25	14.36 ± 6.46	30.63 ± 5.4
Married/widowed/divorced	(111) 27.7	14.02 ± 4.48	14.36 ± 5.91	32.09 ± 4.97
*p*	.096	.998	.014*	
Employment status				
Full time	(153) 38.2	14.27 ± 5.05	14.28 ± 6.6	30.81 ± 5.24
Part time	(50) 12.5	15.6 ± 4.98	15.10 ± 6.02	32.94 ± 5.80
Unemployed	(198) 49.4	14.81 ± 5.09	14.23 ± 6.1	30.72 ± 5.18
*p*	.252	.676	.025**	
Educational level				
Up to elementary school	(11) 2.7	12.45 ± 1.05	14.36 ± 2.01	28,45 ± 1.63
High school	(69) 17.2	11.84 ± 0.47	12.13 ± 0.7	30.10 ± 0.53
Undergraduate degree	(197) 49.1	14.76 ± 0.34	14.45 ± 0.45	30.73 ± 0.37
Graduate degree	(124) 30.9	16.41 ± 0.47	15.45 ± 0.55	32.35 ± 0.50
*p*	<.001***	.01***	.023***	
Total income				
Very low	(95) 23.7	13.50 ± 5.04	13.91 ± 6.86	30.45 ± 5.40
Low	(132) 32.9	14.64 ± 5.04	14.43 ± 5.95	31.29 ± 5.35
Moderate	(114) 28.4	15.13 ± 5.14	14.26 ± 6.38	31.00 ± 5.33
High	(60) 15	15.93 ± 4.7	15.08 ± 6.0	31.45 ± 5.18
*p*	.022**	.729	.612	
BMI				
Underweight	(35) 8.7	14.17 ± 5.17	14.57 ± 7.29	29.02 ± 4.44
Normal weight	(206) 51.2	15.55 ± 5.32	14.36 ± 6.04	31.24 ± 5.75
Overweight	(100) 24.9	13.73 ± 4.63	14.18 ± 6.29	31.12 ± 4.93
Obese	(60) 15	13.71 ± 4.33	4.53 ± 6.75	31.33 ± 4.74
*p*		.007**	.983	.138

*Note*: Age in years, total income by LBP/month, weight status by BMI. As per WHO: underweight (BMI < 18.5 kg/m^2^); normal weight (18.5 ≤ BMI ≤ 24.9 kg/m^2^); overweight (25.0 ≤ BMI ≤ 29.9 kg/m^2^); obese (BMI ≥ 30 kg/m^2^).

Abbreviations: BMI: body mass index in kg/m^2^; LBP: Lebanese pound.

^a^ Awareness scores: poor ≤14, fair: 14.3–19.3, good ≥19.6.

^b^ Knowledge scores: poor ≤16, fair: 16.3–22, good ≥22.4.

^c^ Behavior scores: poor ≤27, fair: 27.5–37.3, good ≥37.8.

*T test for independent samples; **One‐way ANOVA test; ***Nonparametric test independent samples Kruskal–Wallis. *p* < .05 (statistically significant).

### 3.3 Association of TF‐related awareness, knowledge, and behavior scores with sociodemographic characteristics

Regression analysis in Table 3 showed robust relationship association between sociodemographic variables included in the model and awareness, knowledge, and behavior scores (*p* < .001). With other variables controlled for, predicted female awareness, knowledge, and behavior scores were 1.4 times (β = 1.439; *p* = .006; CI: 0.425–2.453), 2.5 times (β = 2.550; *p* < .001; CI: 1.260–3.841), and 2 times (β = 1.923; *p* = .001; CI: 0.786–3.061) higher than males. People aged 35–54 (β = 2.802; *p* = .002, CI: 1.057–4.458) and 55–64 (β = 3.299; *p* = .006, CI: 0.973–5.625) had behavior scores 3 times higher than those aged 18–34. Having a part‐time job increases behavior score 2 times higher than full‐time working (β = 2.239; *p* = .008, CI: 0.580–3.898). Subjects with higher education had an awareness score 3 times higher than participants with intermediate or lower education (β = 3.150; *p* = .040, CI: 0.149–6.152). Having a high school degree (β = 3.933; *p* = .021, CI: 0.594–7.272), a university degree (β = 4.638; *p* = .005, CI: 1.391–7.885), or a higher education (β = 6.367; *p* < .001, CI: 3.052–9.681) had a predicted behavior score 4, 5, and 6 times higher, respectively, than subjects with intermediate or lower education. Those with higher incomes (β = 1.818; *p* = .025, CI: 0.230–3.405) and of normal weight (β = 1.898; *p* = .025, CI: 0.241–3.556) had an awareness score 2 times higher than subjects with lower incomes and were underweight. Normal weight (β = 2.437; *p* = .010, CI: 0.592–4.281), overweight (β = 2.324; *p* = .027, CI: 0.263–4.365), and obese (β = 2.612; *p* = .022, CI: 0.381–4.843) participants had a behavior score 2 times higher than those who were underweight.

**TABLE 3:** Multivariate regression analysis demonstrating the association of awareness, knowledge, and behaviors practices scores with sociodemographic characteristics.

Variables	Awareness score			Knowledge score			Behaviors practices score		
	Unstandardized coefficient, β	*p*	[95% CI]	Unstandardized coefficient, β [95% CI]	*p*	[95% CI]	Unstandardized coefficient, β [95% CI]	*p*	[95% CI]
Gender									
Male	Reference			Reference			Reference		
Female	1.439	.006*	[0.425 to 2.453]	2.55	<.001*	[1.260 to 3.841]	1.923	.001*	[0.786 to 3.061]
Age (years)									
18–34	Reference			–	–	–	Reference		
35–54	−1.511	.057	[−3.068 to 0.045]	–	–	–	2.802	.002*	[1.057 to 4.458]
55–64	−0.905	.414	[−3.081 to 1.270]	–	–	–	3.299	.006*	[0.973 to 5.625]
Employment status									
Full time	–	–	–	–	–	–	Reference		
Part time	–	–	–	–	–	–	2.239	.008*	[0.580 to 3.898]
Unemployed	–	–	–	–	–	–	0.983	.108	[−0.216 to 2.182]
Educational level									
Intermediate or lower	Reference			Reference			Reference		
High school or technical degree	−0.668	.66	[−3.656 to 2.319]	−1.773	.372	[−5.678 to 2.131]	3.933	.021*	[0.594 to 7.272]
University degree	1.512	.309	[−1.409 to 4.434]	0.028	.988	[−3.705 to 3.760]	4.638	.005*	[1.391 to 7.885]
Higher education masters/doctorate	3.15	.04*	[0.149 to 6.152]	0.77	.691	[−3.032 to 4.572]	6.367	<.001*	[3.052 to 9.681]
Total income (LBP/month)									
Very low	Reference			–	–	–	–	–	–
Low	0.605	.338	[−0.636 to 1.847]	–	–	–	–	–	–
Moderate	1.021	.124	[−0.282 to 2.324]	–	–	–	–	–	–
High	1.818	.025*	[0.230 to 3.405]	–	–	–	–	–	–
BMI									
Underweight	Reference						Reference		
Normal weight	1.898	.025*	[0.241 to 3.556]	–	–	–	2.437	.01*	[0.592 to 4.281]
Overweight	1.094	.251	[−0.779 to 2.966]	–	–	–	2.314	.027*	[0.263 to 4.365]
Obese	0.883	.388	[−1.125 to 2.892]	–	–	–	2.612	.022*	[0.381 to 4.843]
Residence									
Beirut	Reference			Reference			–	–	–
North	−0.85	.327	[−2.554 to 0.853]	−2.274	.047*	[−4.522 to −0.026]	–	–	–
South	0.469	.573	[−1.167 to 2.105]	−0.3	.787	[−2.475 to 1.875]	–	–	–
Bekaa	−0.305	.583	[−1.396 to 0.786]	−1.22	.093	[−2.645 to 0.205]	–	–	–

*Note*: Age in years, total income by LBP/month, weight status by BMI. As per WHO: underweight (BMI < 18.5 kg/m^2^); normal weight (18.5 ≤ BMI ≤ 24.9 kg/m^2^); overweight (25.0 ≤ BMI ≤ 29.9 kg/m^2^); obese (BMI ≥ 30 kg/m^2^).

Abbreviations: BMI, body mass index in kg/m^2^; LBP: Lebanese pound.

* *p* < .05 (statistically significant).

## 4 DISCUSSION

This study examined awareness, knowledge, and behaviors related to TFs among adults in Lebanon, a country in the Middle East region with one of the highest rates of cardiovascular diseases (CVDs) worldwide (WHO, 2019b). Our findings suggest that consumer awareness and knowledge about TFs are relatively low, and the majority exhibit fair behavioral practices aimed at reducing TFs intake. In previous literature, awareness was typically assessed by whether participants had heard of TFs (Blitstein & Evans, 2006;Ellis & Glanville, 2010; Jasti & Kovacs, 2010; Nasser et al., 2011). In our study, only 49% of subjects reported having heard of TFs, a much lower percentage compared to earlier studies. For example, Lin and Yen (2010) found that 67% of respondents in Colombia had heard of TFs (Blitstein & Evans, 2006), while Nasser et al. (2011), Ellis and Glanville (2010), and Jasti and Kovacs (2010) reported higher percentages in Canada and the United States (ranging from 92% to 98%) (Ellis & Glanville, 2010; Jasti & Kovacs, 2010; Nasser et al., 2011). Interestingly, despite the close relationship between PHOs and TFs, PHOs had a lower level of consumer awareness than TFs in our study. Only 36% of respondents reported hearing of PHOs, like findings in Saudi Arabia (35%), (Kamel & AL‐Otaibi, 2018) whereas Lin & Yen (2010) found a higher awareness rate with 68% of participants having heard of PHOs (Blitstein & Evans, 2006). These differences may be attributed to the lack of interventions and awareness campaigns regarding TFs and PHOs in the Middle East, particularly in Arab countries (WHO, 2020). Consumers in our study sought information from various sources, with social media being the most used and nutritional facts panels being the least used. The reliance on the Internet may be influenced by the age of participants, as the majority are young adults aged between 18 and 34, who may prefer to access nutrition information in trendy ways rather than traditional sources such as television and print media (Blitstein & Evans, 2006; Quaidoo et al., 2018). Additionally, they may perceive the interpretation provided by social media as more valuable than the information presented on nutrition labels, which they may find difficult to interpret (Quaidoo et al., 2018). Most consumers (86%) reported that they always (21%) or sometimes (65%) look for information on food labels. These percentages are like those reported by Nasser et al. (2011), where 82% of consumers looked for information on food packages while shopping, (Nasser et al., 2011) and higher than findings by Kamel and AL‐Otaibi (2018), where 78% stated not reading food labels (Kamel & AL‐Otaibi, 2018). This suggests that Lebanese adults are generally conscientious about their health but lack awareness specifically about TFs. Gender differences were noted, as reported by Jasti and Kovacs (2010), with males being less likely than females to read food labels or have heard of TFs (Jasti & Kovacs, 2010). Similarly, our study found higher TFs awareness among females, who identified TFs as a major concern and reported hearing about TFs and PHOs more frequently. However, there was no difference in the frequency of looking at food labels between genders. This may be attributed to evolving gender roles, with more men taking on grocery shopping responsibilities (Ellis & Glanville, 2010).

Our study revealed that over half of the participants had poor knowledge scores. Approximately 54% indicated having only a limited understanding of TFs, yet interestingly, 53% correctly identified it as a type of fat, and 81% believed that listing TFs information on food labels is necessary. Nearly all participants recognized TFs as detrimental to health (94%). These results were more pronounced among females, aligning with findings from New York, which underscored a lack of knowledge and use of food labels among college students (Jasti & Kovacs, 2010). Our findings were also consistent with other studies. Pletzke et al. (2010) demonstrated that less than half of participants in Illinois, USA, felt very knowledgeable about TFs before receiving nutrition education (Pletzke et al., 2010), while Nasser et al. (2011) found that a majority (79%) reported having little knowledge (Nasser et al., 2011). Jasti and Kovacs (2010) reported that 61% of participants knew that TFs were a type of fat, slightly higher than the 53% in our study, and 65% were aware of the mandatory listing of TFs on food labels, which is lower than our findings (WHO, 2018f). Similarly, participants in our study demonstrated some level of accuracy in identifying foods containing TFs, with greater knowledge observed among women, particularly regarding processed and manufactured foods, consistent with findings from other studies (Eckel et al., 2009; Jasti & Kovacs, 2010; Nasser et al., 2011). On the contrary, participants in this study were largely unaware of natural and other sources of TFs, such as vegetable shortening, breakfast cereals, and dip/salad dressing. This indicates a knowledge gap regarding TFs sources, consistent with findings from Nasser et al.'s (2011) study, where only a small proportion correctly identified TFs as naturally occurring in milk (Nasser et al., 2011). A significant proportion, particularly among females, correctly associated TFs with negative health outcomes such as elevated levels of bad cholesterol (low‐density lipoprotein, LDL) (76%), hypertension (61%), heart problems (76%), and obesity (79%). These proportions were close but higher than those reported in other studies. Ellis and Glanville (2010) demonstrated that more women than men linked TFs to the risk of obesity (82% vs. 69%) (Ellis & Glanville, 2010). Eckel et al.'s study found that 68% correctly associated TFs with an increased risk of heart disease, (Eckel et al., 2009) while less than half of the participants in Jasti et al.'s study knew that diets high in TFs raise levels of bad cholesterol or LDL (Jasti & Kovacs, 2010). However, Nasser et al. (2011) showed that most respondents correctly identified TFs' effects on raising cholesterol levels and obesity (92%) (Nasser et al., 2011). Approximately half of the participants, especially females, acknowledged to some extent the role of TFs in enhancing food flavor. Similarly, 55% of subjects in Ellis and Glanville (2010) study believed that TFs improve flavor, with no gender difference observed (Ellis & Glanville, 2010). These findings suggest that participants, especially women, were knowledgeable about the negative impacts of TFs. However, knowledge levels regarding food sources of TFs remain insufficient. Women have been reported to possess more nutrition knowledge than males (Kiefer et al., 2005). Their heightened awareness regarding TFs might be due to the portrayal of these fats as “bad” fats in the literature (de Souza et al., 2015; Guasch‐Ferré et al., 2015), directly linked to negative health impacts, especially on heart health (de Souza et al., 2015; Guasch‐Ferré et al., 2015; Wang et al., 2016). This information has been widely promoted and disseminated through social media and the Internet (WHO, 2018f; American Heart Association [AHA], 2007), the primary platforms used for seeking nutrition information (Quaidoo et al., 2018).

Participants exhibited favorable practices regarding the use of different types of fats in cooking. Approximately 59% of subjects, predominantly women, reported low consumption of fried and baked foods, which are among the main sources of TF‐containing foods. However, Jasti and Kovacs (2010) found that over 10% of participants, mainly men, reported high consumption frequency (Jasti & Kovacs, 2010). In our study, while most respondents expressed a willingness to purchase foods labeled as “zero TFs” and occasionally opted for “healthier” menu items, they generally did not actively seek TFs information on food labels. Despite attempting to reduce TF intake and avoiding favorite snacks containing TFs, there was limited evidence of participants altering their food choices by preferring products with lower TF content or completely avoiding those with partially hydrogenated fats. These observations suggest that factors such as price and taste are as crucial as perceived health value in determining food purchasing behaviors. Similarly, Ellis and Glanville (2010) found that while most respondents (81%) were trying to reduce TF intake, few indicated purchasing products with zero TF claims (Ellis & Glanville, 2010). According to Jasti and Kovacs (2010), the prevalence of nonuse of TF information on food labels was relatively high (38%) (Jasti & Kovacs, 2010). Nasser et al. (2011) revealed that although 73% indicated making changes to decrease their intake of TF, 63% stated they would not stop eating snacks with TFs. Additionally, none of the consumers specifically mentioned looking for TFs on food labels (Ellis & Glanville, 2010). Pletzke et al. (2010) discovered that after receiving TF education, participants considered TFs when purchasing foods more frequently (Pletzke et al., 2010). Similarly, a greater number of individuals exhibited altered behaviors after the launch of the American Heart Association's (AHA, 2007) campaign in 2007, such as actively seeking TF information, purchasing products labeled as “zero TF,” scrutinizing TF details before making purchase decisions, and adopting “zero/low TF” versions of solid fat products compared to the previous year (Pletzke et al., 2010). Additionally, Eckel et al. (2009) emphasized the necessity for education to enhance awareness and assist consumers in making informed choices while grocery shopping, cooking at home, and dining out (Eckel et al., 2009). Furthermore, the study highlighted that females demonstrated a greater inclination to inspect food labels for sodium, serving sizes, and partially hydrogenated oils (PHO), displaying improved behaviors concerning cooking oil usage and exhibiting heightened attention to health by reducing consumption of fried or baked foods and high‐fat foods (Eckel et al., 2009). In fact, women have shown to have better diet quality than men, (Ellis & Glanville, 2010) and these findings are consistent with other studies reporting better behavior practices and food label use among women (Ellis & Glanville, 2010; Jasti & Kovacs, 2010; Nasser et al., 2011).

The study showed significant relationships between some of the sociodemographic variables and awareness, knowledge, and behavior scores. According to our findings, being a woman and having higher education level were associated with higher levels of TFs awareness, knowledge, and behavior scores (Tables S1–S3). These results were in accordance with Eckel et al. (2009), who showed that participants with higher knowledge, awareness, and behavior scores were more likely to be females with at least some college education (Eckel et al., 2009). Similarly, Lin and Yen (2010) found higher awareness scores in females with higher education (Lin & Yen, 2010). Differences by gender were also seen in other studies (Ellis & Glanville, 2010; Jasti & Kovacs, 2010; Yahia et al., 2016). However, Hess et al. (2005) showed no association between gender and knowledge scores, but found it highly associated with higher education level (Hess et al., 2005). In fact, educated people are more aware of health importance and are more likely to read nutrition information than their counterparts who may lack comprehensive understanding of health‐related information (Zajacova & Lawrence, 2018). Gender differences may be since, in addition to being more interested in nutrition as previously reported, female's role in the household (cooking and food purchasing) makes them more apt to seek health‐related information than men and improves their dietary behaviors (Cerrato & Cifre, 2018; Vanderlee et al., 2018). No significant difference between age and knowledge scores was reported in our article which was similar to Jasti and Kovacs (2010) and Hess et al. (2005), but in contrast to other studies where older age was significantly related to higher knowledge scores (Blitstein & Evans, 2006; Eckel et al., 2009). On the other hand, participants aged 18–34 reported having the lowest behavior scores in our study which was in accordance with other findings (Eckel et al., 2009; Nasser et al., 2011). However interestingly, this range of age was found to have higher TFs awareness than older aged, in contrast to other studies where participants under age 40 reported lower awareness (Blitstein & Evans, 2006; Jasti & Kovacs, 2010; Nasser et al., 2011). This might be because most participants in this range are educated and more open to information sources thus may be more aware of TFs but show low health engagements. This can be probably explained by other factors affecting their food behaviors such as the expense of healthy food relative to unhealthy food, and lack of time and facilities to plan, shop, prepare, and cook healthy foods (McGowan et al., 2017; Munt et al., 2017).

Our study revealed that being married and working part time were linked to higher behavior scores, whereas previous studies showed a similar association but with knowledge (Hess et al., 2005). Research has indeed shown that being married or living with a partner provides support and encourages individuals to adopt healthier lifestyles, (Dupre & Nelson, 2016; Hess et al., 2005) which could potentially explain our findings. Conversely, no significant difference was found between employment status and knowledge scores in studies conducted by Jasti and Kovacs (2010) and Hess et al. (2005), which aligns with our results (Jasti & Kovacs, 2010; Saadeh et al., 2015). Disparities in employment status might be attributed to individuals with part‐time jobs being more financially secure than nonworkers and having more leisure time than full‐time workers, enabling them to afford and adopt better behavioral practices (Munt et al., 2017).

Additionally, higher income levels were linked to higher awareness scores, consistent with other research findings (Eckel et al., 2009). This could be attributed to the fact that individuals with higher incomes are better equipped to afford high‐quality foods compared to those with lower incomes who may have more limited financial resources (French et al., 2019). Regarding the place of residence, participants from the North region were associated with lower knowledge scores in our study, indicating that residents in this area may not have access to the same resources as those from Beirut or Mount Lebanon, whether in terms of education or economic opportunities. Geographical disparities were also noted by Eckel et al. (2009).

Furthermore, individuals with normal weight were correlated with higher awareness scores compared to others and higher behavior scores compared to those who were underweight. Typically, individuals with normal weight strive to maintain their weight through exercise and adopting appropriate nutritional habits, which could account for their elevated awareness and behavior scores. These findings may be associated with body dissatisfaction, which is commonly experienced among obese and underweight individuals (von Lengerke et al., 2012; Zarychta et al., 2020) and has been linked to unhealthy eating behaviors (Wawrzyniak et al., 2020; Zarychta et al., 2020), thus making individuals more susceptible to consuming unhealthy, energy‐dense foods such as fatty and sweet foods.

### 4.1 Strengths and limitations

This study has several limitations. Although the questionnaire was distributed nationwide in Lebanon, certain regions and populations were underrepresented, particularly the North and South regions. Moreover, there was an overrepresentation of younger adults (aged 18–34) in the study sample at the expense of those aged 34 and older. Additionally, the survey was conducted online, potentially contributing to the lower participation rate among older individuals who may be less familiar with using electronic devices. Furthermore, most participants were female (68% female vs. 32% male), indicating that our sample was not randomly selected. Similarly, there was a higher proportion of participants with university degrees or higher education compared to those with lower educational attainment. This discrepancy may be due to the prevalence of university education in Beirut and Mount Lebanon, where most participants resided, compared to other regions in the country. Additionally, individuals with higher educational levels may have been more likely to participate in the survey. The study did not utilize a validated questionnaire due to the unavailability of such a tool; however, the questionnaire was designed based on previous surveys (Eckel et al., 2009; Ellis & Glanville, 2010; Lin & Yen, 2010; Nasser et al., 2011; Pletzke et al., 2010) and underwent pretesting. Furthermore, the survey relied on self‐reported behavior, which may not accurately reflect actual behavior. Obtaining insights into actual consumer behavior would require shadowing and direct observation. Despite these limitations, this study is the first in Lebanon to provide valuable insights into TF‐related awareness, knowledge, and behaviors among Lebanese adults, offering essential information to guide the development of effective, evidence‐based TFs reduction programs and inform appropriate policies.

## 5 CONCLUSION

This study sheds light on specific gaps in TF‐related awareness, knowledge, and behavioral practices among a sample of Lebanese adults, along with variations based on sociodemographic factors. Our findings revealed that while 49% of participants were aware of TFs, a lower proportion (36%) had heard of PHOs. Approximately 54% reported having only a basic understanding of TFs, with the majority demonstrating poor knowledge of TFs food sources, and 44% expressing a reluctance to stop consuming their favorite snacks if they contained TFs. These results suggest that consumer awareness and knowledge regarding TFs are relatively low, with the majority exhibiting moderate behavioral practices aimed at reducing TFs intake. Moreover, being female and having a higher level of education were significantly associated with greater TFs awareness, knowledge, and behavioral scores. Older participants, married individuals, and those with part‐time jobs exhibited higher behavioral scores, while higher income and normal weight were linked to increased awareness scores. Nutrition education holds promise for fostering behavioral changes related to TFs intake, underscoring the importance of targeted education campaigns focusing on TFs, their dietary sources, and their impact on health.

This study represents the first of its kind in the region to explore TFs and societal behaviors toward such foods. It underscores the knowledge deficit in a developing country like Lebanon, particularly concerning public awareness and education, which is crucial given the prevalence of CVDs as the leading cause of death in the country. Despite global initiatives to eliminate TFs from the food supply, efforts in Lebanon remain limited. Our findings have significant public health implications, highlighting the importance of targeting specific demographics, such as age and gender, with tailored education campaigns to improve awareness, knowledge, and behaviors regarding TFs. By leveraging this information, targeted populations can play a pivotal role in raising awareness about the dangers of TFs within their communities.

Moreover, there is a pressing need to enhance awareness and knowledge among Lebanese youth, particularly males, regarding TFs content in foods. This can be achieved through educational initiatives focused on educating youth about the nutritional risks associated with TFs consumption, incentivizing the reduction of high TFs food sales on university campuses, and promoting the availability of healthier, lower TFs options for students. Nutrition sessions led by health educators should also address the topic of TFs comprehensively. Community‐based interventions and environmental support involving health professionals, educators, media, and local authorities are recommended to facilitate sustainable changes in awareness, knowledge, and behavior related to TFs consumption. The findings of this study should serve as a foundation for the development of intervention strategies aimed at enhancing TF‐related knowledge in Lebanon, as well as informing the development of policies tailored to the Arab region.

## AUTHOR CONTRIBUTIONS

**Marianne El Hajj:** Conceptualization (lead); data curation (lead); formal analysis (lead); funding acquisition (lead); investigation (lead); methodology (lead); project administration (lead); resources (lead); software (lead); supervision (supporting); validation (supporting); visualization (supporting); writing – original draft (lead); writing – review and editing (supporting). **Jennifer Abou Chaaya:** Conceptualization (lead); data curation (lead); formal analysis (lead); funding acquisition (lead); investigation (lead); methodology (lead); project administration (lead); resources (lead); software (lead); supervision (supporting); validation (supporting); visualization (supporting); writing – original draft (lead); writing – review and editing (supporting). **Jessica Abou Chaaya:** Supervision (supporting); validation (supporting); visualization (supporting); writing – original draft (supporting); writing – review and editing (equal). **Maya Tueni:** Conceptualization (supporting); data curation (supporting); formal analysis (supporting); funding acquisition (supporting); investigation (supporting); methodology (supporting); project administration (supporting); resources (supporting); software (supporting); supervision (lead); validation (lead); visualization (lead); writing – original draft (supporting); writing – review and editing (lead).

## CONFLICT OF INTEREST STATEMENT

All authors declared that there are no conflicts of interest.

## ETHICS STATEMENT AND CONSENT TO PARTICIPATE

All participants signed a written informed consent to participate, and they were given the option to withdraw as per their convenience. All participants' data and information were kept confidential.

## CONSENT FOR PUBLICATION

All participants signed an electronic informed consent to use their data in a confidential manner.

## Supporting information


Data S1.

## Data Availability

The data that support the findings of this study are available from the first two coauthors Jennifer Abou Chaaya and Marianne El Hajj upon reasonable request.

## References

[fsn34211-bib-0001] American Heart Association [AHA] . (2007). *Trans fats* [Internet]. https://www.heart.org/en/healthy‐living/healthy‐eating/eat‐smart/fats/trans‐fat

[fsn34211-bib-0002] Blitstein, J. L. , & Evans, W. D. (2006). Use of nutrition facts panels among adults who make household food purchasing decisions. Journal of Nutrition Education and Behavior, 38(6), 360–364. 10.1016/j.jneb.2006.02.009 17142192

[fsn34211-bib-0003] Brouwer, I. A. , Wanders, A. J. , & Katan, M. B. (2010). Effect of animal and industrial trans fatty acids on HDL and LDL cholesterol levels in humans—A quantitative review. PLoS One, 5(3), e9434. 10.1371/journal.pone.0009434 20209147 PMC2830458

[fsn34211-bib-0004] Cerrato, J. , & Cifre, E. (2018). Gender inequality in household chores and work‐family conflict. Frontiers in Psychology, 9, 1330. 10.3389/fpsyg.2018.01330 30123153 PMC6086200

[fsn34211-bib-0005] de Souza, R. J. , Mente, A. , Maroleanu, A. , Cozma, A. I. , Ha, V. , Kishibe, T. , Uleryk, E. , Budylowski, P. , Schünemann, H. , Beyene, J. , & Anand, S. S. (2015). Intake of saturated and trans unsaturated fatty acids and risk of all cause mortality, cardiovascular disease, and type 2 diabetes: Systematic review and meta‐analysis of observational studies. The BMJ, 351, h3978. 10.1136/bmj.h3978 26268692 PMC4532752

[fsn34211-bib-0006] Dupre, M. E. , & Nelson, A. (2016). Marital history and survival after a heart attack. Social Science & Medicine, 170, 114–123. 10.1016/j.socscimed.2016.10.013 27770749 PMC5127274

[fsn34211-bib-0007] Eckel, R. H. , Kris‐Etherton, P. , Lichtenstein, A. H. , Wylie‐Rosett, J. , Groom, A. , Stitzel, K. F. , & Yin‐Piazza, S. (2009). Americans' awareness, knowledge, and behaviors regarding fats: 2006‐2007. Journal of the American Dietetic Association, 109(2), 288–296. 10.1016/j.jada.2008.10.048 19167956

[fsn34211-bib-0008] Ellis, S. , & Glanville, N. T. (2010). Trans fat information on food labels: Consumer use and interpretation. Canadian Journal of Dietetic Practice and Research, 71(1), 6–10. 10.3148/71.1.2010.6 20205970

[fsn34211-bib-0009] Farhat, A. , Jaalouk, D. E. , Moukarzel, S. , & Ayoub, J. (2016). Consumption of trans fatty acid and omega 6 to omega 3 ratio in Lebanese adults. Nutrition & Food Science, 46, 120–129. 10.1108/NFS-07-2015-0089

[fsn34211-bib-0010] French, S. A. , Tangney, C. C. , Crane, M. M. , Wang, Y. , & Appelhans, B. M. (2019). Nutrition quality of food purchases varies by household income: The SHoPPER study. BMC Public Health, 19(1), 231. 10.1186/s12889-019-6546-2 30808311 PMC6390355

[fsn34211-bib-0011] Guasch‐Ferré, M. , Babio, N. , Martínez‐González, M. A. , Corella, D. , Ros, E. , Martín‐Peláez, S. , Estruch, R. , Arós, F. , Gómez‐Gracia, E. , Fiol, M. , Santos‐Lozano, J. M. , Serra‐Majem, L. , Bulló, M. , Toledo, E. , Barragán, R. , Fitó, M. , Gea, A. , Salas‐Salvadó, J. , & PREDIMED Study Investigators . (2015). Dietary fat intake and risk of cardiovascular disease and all‐cause mortality in a population at high risk of cardiovascular disease. The American Journal of Clinical Nutrition, 102(6), 1563–1573. 10.3945/ajcn.115.116046 26561617

[fsn34211-bib-0012] Harvard T.H. Chan School of Public Health . (2018). Artificial trans fats banned in U.S. [Internet]. *News*. https://www.hsph.harvard.edu/news/hsph‐in‐the‐news/us‐bans‐artificial‐trans‐fats/

[fsn34211-bib-0013] Hess, S. , Yanes, M. , Jourdan, P. , & Edelstein, S. (2005). Trans fat knowledge is related to education level and nutrition facts label use in health‐conscious adults. Topics in Clinical Nutrition, 20, 109–117. 10.1097/00008486-200504000-00004

[fsn34211-bib-0014] Hoteit, M. , Zoghbi, E. , Rady, A. , Shankiti, I. , Ibrahim, C. , & Al‐Jawaldeh, A. (2021a). Assessment of industrially produced trans fatty acids in traditional dishes, Arabic sweets, and market food products and its risks on non‐communicable diseases in Lebanon. Frontiers in Nutrition, 8, 727548. 10.3389/fnut.2021.727548 34746203 PMC8566673

[fsn34211-bib-0015] Hoteit, M. , Zoghbi, E. , Rady, A. , Shankiti, I. , Ibrahim, C. , & Al‐Jawaldeh, A. (2021b). Non‐conjugated‐industrially‐produced‐trans fatty in Lebanese foods: The case of Elaidic and Linolelaidic acids. Nutrients, 13(10), 3664. 10.3390/nu13103664 34684664 PMC8536972

[fsn34211-bib-0016] Jasti, S. , & Kovacs, S. (2010). Use of trans fat information on food labels and its determinants in a multiethnic college student population. Journal of Nutrition Education and Behavior, 42(5), 307–314. 10.1016/j.jneb.2009.06.004 20637701

[fsn34211-bib-0017] Jawaldeh, A. A. , & Al‐Jawaldeh, H. (2018). Fat intake reduction strategies among children and adults to eliminate obesity and non‐communicable diseases in the eastern Mediterranean region. Children, 5(7), E89. 10.3390/children5070089 PMC6069461 29966315

[fsn34211-bib-0018] Kamel, S. , & AL‐Otaibi, H. (2018). Trans‐fats declaration, awareness and consumption in Saudi Arabia. Current Nutrition & Food Science, 6, 748–756. 10.12944/CRNFSJ.6.3.17

[fsn34211-bib-0019] Kiefer, I. , Rathmanner, T. , & Kunze, M. (2005). Eating and dieting differences in men and women. The Journal of Men's Health & Gender, 2(2), 194–201. 10.1016/j.jmhg.2005.04.010

[fsn34211-bib-0020] Lichtenstein, A. H. (2016). Fatty acids: Trans fatty acids. In Encyclopedia of food and health (pp. 645–648). Elsevier. 10.1016/B978-0-12-384947-2.00280-4

[fsn34211-bib-0021] Lin, C.‐T. J. , & Yen, S. T. (2010). Knowledge of dietary fats among US consumers. Journal of the American Dietetic Association, 110(4), 613–618. 10.1016/j.jada.2009.12.020 20338288

[fsn34211-bib-0022] Liu, L. , Liu, Y.‐P. , Wang, J. , An, L.‐W. , & Jiao, J.‐M. (2016). Use of a knowledge‐attitude‐behaviour education programme for Chinese adults undergoing maintenance haemodialysis: Randomized controlled trial. The Journal of International Medical Research, 44(3), 557–568. 10.1177/0300060515604980 26951842 PMC5536721

[fsn34211-bib-0023] McGowan, L. , Caraher, M. , Raats, M. , Lavelle, F. , Hollywood, L. , McDowell, D. , Spence, M. , McCloat, A. , Mooney, E. , & Dean, M. (2017). Domestic cooking and food skills: A review. Critical Reviews in Food Science and Nutrition, 57(11), 2412–2431. 10.1080/10408398.2015.1072495 26618407

[fsn34211-bib-0024] Micha, R. , Khatibzadeh, S. , Shi, P. , Fahimi, S. , Lim, S. , Andrews, K. G. , Engell, R. E. , Powles, J. , Ezzati, M. , Mozaffarian, D. , & Global Burden of Diseases Nutrition and Chronic Diseases Expert Group NutriCoDE . (2014). Global, regional, and national consumption levels of dietary fats and oils in 1990 and 2010: A systematic analysis including 266 country‐specific nutrition surveys. The BMJ, 348, g2272. 10.1136/bmj.g2272 24736206 PMC3987052

[fsn34211-bib-0025] Munt, A. E. , Partridge, S. R. , & Allman‐Farinelli, M. (2017). The barriers and enablers of healthy eating among young adults: A missing piece of the obesity puzzle: A scoping review. Obesity Reviews, 18(1), 1–17. 10.1111/obr.12472 27764897

[fsn34211-bib-0026] Nasser, R. , Cook, S. , Bashutski, M. , Hill, K. , Norton, D. , Coleman, J. , Walker, S. , & Charlebois, S. (2011). Consumer perceptions of trans fats in 2009 show awareness of negative effects but limited concern regarding use in snack foods. Applied Physiology, Nutrition, and Metabolism, 36(4), 526–532. 10.1139/h11-045 21854161

[fsn34211-bib-0027] Pletzke, V. , Henry, B. , Ozier, A. , & Umoren, J. (2010). The effect of nutrition education on knowledge, attitude, and behavior relating to trans fatty acids in foods. Family and Consumer Sciences Research Journal, 39, 173–183. 10.1111/j.1552-3934.2010.02055.x

[fsn34211-bib-0028] Quaidoo, E. Y. , Ohemeng, A. , & Amankwah‐Poku, M. (2018). Sources of nutrition information and level of nutrition knowledge among young adults in the Accra metropolis. BMC Public Health, 18(1), 1323. 10.1186/s12889-018-6159-1 30497442 PMC6267800

[fsn34211-bib-0029] Saadeh, C. , Toufeili, I. , Zuheir Habbal, M. , & Nasreddine, L. (2015). Fatty acid composition including trans‐fatty acids in selected cereal‐based baked snacks from Lebanon. Journal of Food Composition and Analysis, 41, 81–85. 10.1016/j.jfca.2015.01.014

[fsn34211-bib-0030] Vanderlee, L. , Hobin, E. P. , White, C. M. , & Hammond, D. (2018). Grocery shopping, dinner preparation, and dietary habits among adolescents and young adults in Canada. Canadian Journal of Dietetic Practice and Research, 79(4), 157–163. 10.3148/cjdpr-2018-025 30280918

[fsn34211-bib-0031] von Lengerke, T. , Mielck, A. , & KORA Study Group . (2012). Body weight dissatisfaction by socioeconomic status among obese, preobese and normal weight women and men: Results of the cross‐sectional KORA Augsburg S4 population survey. BMC Public Health, 12, 342. 10.1186/1471-2458-12-342 22571239 PMC3533751

[fsn34211-bib-0032] Wang, Q. , Afshin, A. , Yakoob, M. Y. , Singh, G. M. , Rehm, C. D. , Khatibzadeh, S. , Micha, R. , Shi, P. , Mozaffarian, D. , & Global Burden of Diseases Nutrition and Chronic Diseases Expert Group (NutriCoDE) . (2016). Impact of nonoptimal intakes of saturated, polyunsaturated, and trans fat on global burdens of coronary heart disease. Journal of the American Heart Association, 5(1), e002891. 10.1161/JAHA.115.002891 26790695 PMC4859401

[fsn34211-bib-0033] Wawrzyniak, A. , Myszkowska‐Ryciak, J. , Harton, A. , Lange, E. , Laskowski, W. , Hamulka, J. , & Gajewska, D. (2020). Dissatisfaction with body weight among polish adolescents is related to unhealthy dietary behaviors. Nutrients, 12(9), E2658. 10.3390/nu12092658 PMC7551787 32878216

[fsn34211-bib-0034] World Health Organization [WHO] . (2018a). *Nutrition: Trans fat* [Internet]. https://www.who.int/news‐room/questions‐and‐answers/item/nutrition‐trans‐fat

[fsn34211-bib-0035] World Health Organization [WHO] . (2018b). *WHO plan to eliminate industrially‐produced trans‐fatty acids from global food supply* [Internet]. https://www.who.int/news/item/14‐05‐2018‐who‐plan‐to‐eliminate‐industrially‐produced‐trans‐fatty‐acids‐from‐global‐food‐supply

[fsn34211-bib-0036] World Health Organization [WHO] . (2018c). *Draft guidelines: Saturated fatty acid and trans‐fatty acid intake for adults and children* [Internet]. https://www.draft‐who‐sfa‐tfa‐guidelines‐public‐consultation.pdf 37490572

[fsn34211-bib-0037] World Health Organization [WHO] . (2018d). *Noncommunicable diseases country profiles 2018* [Internet]. https://www.who.int/publications‐detail‐redirect/9789241514620

[fsn34211-bib-0042] World Health Organization [WHO] . (2018e). *REPLACE trans fat* [Internet]. https://www.who.int/teams/nutrition‐and‐food‐safety/replace‐trans‐fat

[fsn34211-bib-0043] World Health Organization [WHO] . (2018f). *Sample media action plan for trans fat Public Service Announcement (PSA)* [Internet]. https://c‐example‐media‐action‐plan‐for‐psa.pdf

[fsn34211-bib-0038] World Health Organization [WHO] . (2019a). *REPLACE trans fat: An action package to eliminate industrially produced trans‐fatty acids: Module 2: Promote: How‐to guide for determining the best replacement oils and interventions to promote their use* [Internet]. Report No.: WHO/NMH/NHD/19.13. https://apps.who.int/iris/handle/10665/324821

[fsn34211-bib-0039] World Health Organization [WHO] . (2019b). *Regional Office for the Eastern Mediterranean. Strategy on nutrition for the eastern Mediterranean region 2020–2030* [Internet]. World Health Organization. Regional Office for the Eastern Mediterranean; 64 p. https://apps.who.int/iris/handle/10665/330059

[fsn34211-bib-0040] World Health Organization [WHO] . (2019c). REPLACE trans fat an action package to eliminate industrially‐produced trans fatty acids . https://cdn.who.int/media/docs/default‐source/nutritionlibrary/replace‐transfat/1‐replace‐framework‐updated‐june‐2019‐ke.pdf?sfvrsn=47e47367_2

[fsn34211-bib-0041] World Health Organization [WHO] . (2020). *Countdown to 2023: WHO report on global trans‐fat elimination 2020* [Internet]. https://www.who.int/publications‐detail‐redirect/9789240010178

[fsn34211-bib-0044] Yahia, N. , Brown, C. A. , Rapley, M. , & Chung, M. (2016). Level of nutrition knowledge and its association with fat consumption among college students. BMC Public Health, 16(1), 1047. 10.1186/s12889-016-3728-z 27716127 PMC5050673

[fsn34211-bib-0045] Zajacova, A. , & Lawrence, E. M. (2018). The relationship between education and health: Reducing disparities through a contextual approach. Annual Review of Public Health, 39, 273–289. 10.1146/annurev-publhealth-031816-044628 PMC5880718 29328865

[fsn34211-bib-0046] Zarychta, K. , Chan, C. K. Y. , Kruk, M. , & Luszczynska, A. (2020). Body satisfaction and body weight in under‐ and healthy‐weight adolescents: Mediating effects of restrictive dieting, healthy and unhealthy food intake. Eating and Weight Disorders: EWD, 25(1), 41–50. 10.1007/s40519-018-0496-z 29520585 PMC6997259

