# Supplementary material for: Awareness, knowledge, and behaviors regarding trans unsaturated fatty acids in a sample of Lebanese adults
Source: Food Sci Nutr. 2024 May 19;12(8):5694–707. doi: 10.1002/fsn3.4211 (PMC11317711; doi:10.1002/fsn3.4211)
Supplement: Supplementary file 1 — Data S1. [file FSN3-12-5694-s001.doc]

**Supplementary Material**

**Appendix 1:**

**Consent Form**

Dear participants,

You are invited to participate in this scientific study. Please take your time to read the following information before confirming your participation in this study. Do not hesitate to ask questions about the questionnaire and the study in general. We thank you for your interest and your attention and we await your participation.

**Study title:** Awareness, knowledge and behaviors regarding trans among a sample of Lebanese adults.
**Study objective:** This is a study of adults consumers in Lebanon, which aims to:

✓ To provide estimates of trans fat awareness, knowledge and behaviors in a sample of Lebanese consumers aged 18–64 years

✓ Investigate the association of socio-demographic factors, knowledge and awareness with self-reported trans-fats related behaviors.

**Principal investigators:** Marianne Al HAJJ & Jennifer ABOU CHAAYA under the supervision of Dr. Maya TUENI for the fulfillment of their master’s studies in human nutrition.

**Address:** Lebanese University, Faculty of Public Health, section 2, Department of Nutrition and Dietetics.

**Location of intended survey sampling:** Online survey.

**Study description:** This is a research study to assess awareness, knowledge and behaviors regarding trans fats in a sample of Lebanese adults. Participation in this study involves completion of a survey comprised of the following 2 parts to be honestly filled out by the participant.

**PART 1:**  PERSONAL DATA CONTAINING THE FOLLOWING SECTIONS:
***SECTION 1:*** *SOCIO-DEMOGRAPHIC INFORMATION*
***SECTION 2:*** *ANTHROPOMETRIC MEASUREMENTS*
***SECTION 3:*** *GENERAL HEALTH QUESTIONS*
**PART 2:** TRANS FAT CONTAINING THE FOLLOWING SECTIONS:
***SECTION 1:*** *TRANS FAT AWARENESS*
***SECTION 2:*** *TRANS FAT KNOWLEDGE*
***SECTION 3:*** *TRANS FAT BEHAVIORS*

**Duration:** The expected duration on this procedure will last approximately 15 minutes

**Risk:** No risk or discomforts are anticipated from taking part in this study.

**Confidentiality:** The information gathered will be kept completely confidential. Only the researchers will have access to the study data and information. You will be assigned a code number which will protect your identity and the key to the code will be kept secret. Your names and identifying details will never be revealed in any publication of the results collated and analyzed.

Please read each question carefully and select the answer that best describes your experience; which you think is most appropriate for your specific situation. Please respond truthfully by checking the boxes you choose with an X and filling in the blank (where there are blank lines/spaces).

The questionnaire is a screening tool and is not intended to provide a diagnosis which can only be made by a certified clinician.

**Investigator approval:** I explained the study in detail to the participant, its nature, its objectives, and I answered all the questions clearly.
***Investigator*** ***name:*** Dr Maya Tueni , Jennifer Abou Chaaya, Marianne Al Hajj

***Investigator signature:***

***Date: ___________________2021***

**Participant’s approval:** I._________________________________ freely agree to participate in this study. I understand that I am free to refuse to answer any question and to withdraw from the study at any time. I understand that my responses will be kept anonymous.

***Participant’s signature:* ________________**

***Date: ___________________2021***

**Appendix 2:**

**Survey**

1. **Personal Data**

**1. Socio – economic and demographic background:**

1) Gender:

❑ Male 1

❑ Female 2

2) Age (years)

❑ 18-24 1

❑ 25-34 2

❑ 35-44 3

❑ 45-54 4

❑ 55-64 5

3) Residence :

❑ Beirut 1

❑ Mount Lebanon 2

❑ North Lebanon 3

❑ South Lebanon / Nabatieh 4

❑ Bekaa 5

4) Marital Status:

❑ Single 1

❑ Married / Widowed / Divorced 2

5) Employment status:

❑ Full-time 1

❑ Part-time 2

❑ Unemployed 3

6) Educational level:

❑ Intermediate or Lower 1

❑ High school or Technical degree 2

❑ University degree 3

❑ Graduate education: Masters/Doctorate 4

7) Number of people currently living at home including you & members/relatives living with you on permanent basis:

❑ 1-3 1

❑ 4-6 2

❑ >6 3

8) How many rooms are there in your home? (Excluding kitchen, hallways bathrooms, balconies):

❑ <4 1

❑ 4-5 2

❑ >5 3

9) What is your total income? (L.L):

❑ Less than 750 000 L. L/month 1

❑ 750 000 – 2 000 000 L.L/month 2

❑ 2 000 000 – 4 000 000 L.L/month 3

❑ More than 4 000 000 L.L /month 4

10) Most meals are consumed at:

❑ Home 1

❑ Restaurants 2

**2. Anthropometric measurements:**

11) Weight (kg):

____________

12) Height (cm):

____________

**3. General health questions:**

13) How would you rate your current state of health?

❑ Excellent 1

❑ Very good 2

❑ Good 3

❑ Fair 4

❑ Poor 5

14) Do you have a specific health problem?

❑ No problem1

❑ High blood pressure 2

❑ Diabetes 3

❑ High cholesterol 4

❑ Heart disease 5

❑ Kidney disease 6

❑ Obesity 7

❑ Cancer 8

❑ Other: ______ 9

1. **Trans Fat**

1. **Trans-fat awareness:**

15) Please check all the types of fats and oils that you have ever heard of:

❑ Vegetable oils

❑ Saturated fats

❑ Trans fats

❑ Animal fats

❑ n-3 fatty acids

❑ Polyunsaturated fats

❑ Monounsaturated fats

❑ Partially hydrogenated oils

16) Have you ever heard of or read about trans fats prior to the survey?

❑ Yes

❑ No

17) If Yes, please specify the source of information:

❑ Television

❑ Internet (social media)

❑ Nutrition Facts panel

❑ Advertisements

❑ Physicians or other health care professionals

❑ Family or friends

❑ Cookbooks

❑ Other sources: ___________

18) What comes the most to your mind when thinking about trans fat?

❑ Unhealthy

❑ Bad fats

❑ Weight gain

❑ Heart disease

❑ Cholesterol

❑ Death

❑ Other: _________

19) Is trans fat a major concern, minor concern, or of no concern to you?

❑ Major concern

❑ Minor concern

❑ No concern

20) What do you base decision on when buying food?

❑ Nutrition value

❑ Price

❑ Need

❑ Likes

❑ Freshness

❑ Quality

❑ Taste

❑ Other: ______

21) When you grocery shop, how often do you look on food label information?

❑ Always

❑ Sometimes

❑ Never

1. **Trans Fat Knowledge:**

22) How much would you say you know about trans fats?

❑ A lot

❑ A little

❑ Nothing

23) What are trans fats (choose 1 correct answer):

❑ A type of fat

❑ A good fat

❑ Cholesterol

❑ I don’t know

24) Are trans fats good for health?

❑ Yes

❑ No

25) Reducing trans fat intake will improve overall health:

❑ Yes

❑ No

❑ I don’t know

26) Do you think listing of trans fat information on food labels is mandatory?

❑ Yes

❑ No

27) Which of these foods, if any, contain trans fats? Check all that apply

❑ French Fries

❑ Lard

❑ Butter

❑ Non-Hydrogenated margarine

❑ Hard margarine

❑ Vegetable shortening

❑ Doughnuts

❑ Candies and desserts

❑ Pastries/Bakery products

❑ Cookies

❑ Crackers

❑ Breakfast cereals

❑ Poultry/Eggs

❑ Meat products

❑ Seafoods

❑ Milk and dairy products

❑ Processed meats

❑ Processed foods

❑ Dip/salad dressing

❑ None of the above

28) Do you agree with these statements?

|  | Yes | No | I don’t know |
| --- | --- | --- | --- |
| Trans fat should be limited |  |  |  |
| Trans fat improves the taste of food |  |  |  |
| Trans fat may increase bad cholesterol (low-density lipoprotein (LDL)) |  |  |  |
| Trans fat intake may increase hypertension |  |  |  |
| Trans intake may increase diabetes |  |  |  |
| Trans fat may increase risk of obesity |  |  |  |
| Trans fat is bad for heart health |  |  |  |

1. **Trans fat behaviors:**

29) Which of the following specific types of information that relate to **heart health** do you look for on food or beverage labels or packages? Please check all that apply.

❑ Sodium

❑ Total fats

❑ Total calories

❑ Cholesterol

❑ Trans fats

❑ Saturated fats

❑ Serving size

❑ Statement about heart health benefits

❑ Partially hydrogenated oils

❑ Hydrogenated oils

❑ None of the above

30) Are you trying to consume less trans fats?

❑ Yes

❑ No

31) Would you stop eating your favorite snack food if you knew it had trans fat in it?

❑ Yes

❑ No

32) How many times in a week do you eat fried foods and baked goods such as fries, doughnuts, pastries, cakes, and cookies?

❑ 0 times/week

❑ 1-2 times/week

❑ 3-4 times/week

❑ 5 times / week

❑ >5 times /week.

33) How often do you use cooking fats in meal preparation?

|  | Never or rarely | Sometimes | Usually or always |
| --- | --- | --- | --- |
| Canola oil |  |  |  |
| Palm oil |  |  |  |
| Olive oil |  |  |  |
| Corn oil |  |  |  |
| Sunflower oil |  |  |  |
| Cooking margarine |  |  |  |
| Butter |  |  |  |
| Lard, pork |  |  |  |

34) Have you changed your food purchases to reduce your trans fat intake?

❑ Yes

❑ No

35) Have you purchased foods labelled “0 trans fat”?

❑ Yes

❑ No

36) Have you read Nutrition Facts panels to choose foods with a low trans fat content?

❑ Yes

❑ No

37) Have you reduced your intake of high fat foods?

❑ Yes

❑ No

38) Have you read ingredients lists to select foods with no partially hydrogenated fats?

❑ Yes

❑ No

39) How often, if at all do you do the following when you are at a restaurant:

|  | Never or Rarely | Sometimes | Usually or Always |
| --- | --- | --- | --- |
| Ask the server about the type of oils or fat used to prepare a food |  |  |  |
| Request ingredient or nutrition information of items on the menu |  |  |  |
| Ask the server to suggest meal option |  |  |  |
| Order an item on the menu because it was marked as being healthier in some way |  |  |  |

Table 1: Awareness related to trans-fat in a sample of Lebanese adults (n=401) by gender

| **Awareness** | **Total *n (%)*** | **Gender** | | ***P-Value* *** |
| --- | --- | --- | --- | --- |
|  |  | Males *n* (%) | Females *n* (%) |  |
| **Types of fats heard of** |  |  |  |  |
| **Have you ever heard of vegetable fat? (yes)** | 365(91.0) | 117(90.0) | 248(91.5) | 0.62 |
| **Have you ever heard of saturated fat? (yes)** | 278(69.3) | 82(63.1) | 196(72.3) | 0.06 |
| **Have you ever heard of trans fat? (yes)** | 198(49.4) | 52(40.0) | 146(53.9) | 0.009** |
| **Have you ever heard of animal fat? (yes)** | 260(64.8) | 80(61.5) | 180(66.4) | 0.338 |
| **Have you ever heard of n-3 fatty acids? (yes)** | 230(57.4) | 65(50.0) | 165(60.9) | 0.039** |
| **Have you ever heard of PUFA fats? (yes)** | 126(31.4) | 33(25.4) | 93(34.3) | 0.071 |
| **Have you ever heard of MUFA? (yes)** | 122(30.4) | 37(28.5) | 85(31.4) | 0.554 |
| **Have you ever heard of PHOs? (yes)** | 146(36.4) | 36(27.7) | 110(40.6) | 0.012** |
| **Source of information ^a^** |  |  |  |  |
| **Television** | 11(4.9) | 10(6.0) | 1(1.7) | 0.237 |
| **Internet (social media)** | 86(38.1) | 24(40.0) | 62(37.3) |  |
| **Nutrition Facts panel** | 21(9.3) | 6(10.0) | 15(9.0) |  |
| **Physicians or other health care professionals** | 38(16.8) | 6(10.0) | 32(19.3) |  |
| **Other sources** | 70(31.0) | 23(38.3) | 47(28.3) |  |
| **What comes to your mind the most when thinking about trans-fat?** |  |  |  |  |
| **Unhealthy** | 198(49.4) | 69(53.1) | 129(47.6) | 0.286 |
| **Bad fats** | 86(21.4) | 24(18.5) | 62(22.9) |  |
| **Weight gain** | 41(10.2) | 16(12.3) | 25(9.2) |  |
| **Heart disease** | 24(6.0) | 6(4.6) | 18(6.6) |  |
| **Cholesterol** | 31(7.7) | 6(4.6) | 25(9.2) |  |
| **Other** | 21(5.2) | 9(6.9) | 12(4.4) |  |
| **Is trans-fat a major concern, minor concern, or of no concern to you?** |  |  |  |  |
| **Major concern** | 165(41.1) | 41(31.5) | 124(45.8) | 0.012** |
| **Minor concern** | 182(45.4) | 65(50.0) | 117(43.2) |  |

| **No concern** | **54(13.5)** | **24(18.5)** | **30(11.1)** |  |
| --- | --- | --- | --- | --- |
| **When you grocery shop, how often do you look on food label information?** |  |  |  |  |
| **Always** | 85(21.2) | 20(15.4) | 65(24.0) | 0.105 |
| **Sometimes** | 260(64.8) | 88(67.7) | 172(63.5) |  |
| **Never** | 56(14.0) | 22(16.9) | 34(12.5) |  |
| **What do you base decision on when buying food?^a^** |  |  |  |  |
| **Nutrition value** | 156(39.0) | 50(38.5) | 106(39.1) | 0.9 |
| **Price** | 189(47.3) | 64(49.2) | 125(46.1) | 0.56 |
| **Need** | 214(53.5) | 62(47.7) | 152(56.1) | 0.115 |
| **Likes** | 144(56.0) | 45(34.6) | 99(36.5) | 0.708 |
| **Freshness** | 202(50.5) | 66(50.8) | 136(50.2) | 0.913 |
| **Quality** | 271(67.8) | 83(63.8) | 188(69.4) | 0.268 |
| **Taste** | 205(51.2) | 72(55.4) | 133(49.1) | 0.237 |
| **Other things** | 12(3.0) | 4(3.1) | 8(3.0) | 0.945 |

*Chi-Square Test; **Significant p-value<0.05; PUFA: Poly-unsaturated fatty acids; MUFA: Mono-unsaturated fatty acids; PHOs: Partially hydrogenated oils. ^a^: Multiple choices were possible, hence total may exceed 100%

Table 2: Knowledge related to trans-fat in a sample of Lebanese adults (n=401) by gender

| **Knowledge** | **Total *n (%)*** | **Gender** | | ***P-Value* *** |
| --- | --- | --- | --- | --- |
|  |  | Males *n* (%) | Females *n* (%) |  |
| **How much would you say you know about trans fats?** |  |  |  |  |
| **A lot** | 49 (12.2) | 13(10.0) | 36(13.3) | 0.005** |
| **A little** | 215 (53.6) | 58(44.6) | 157(57.9) |  |
| **Nothing** | 137 (34.2) | 58(45.4) | 78(28.8) |  |
| **What are trans fats?** |  |  |  |  |
| **A type of fat** | 211 (52.6) | 50(38.5) | 161(59.4) | <0.001** |
| **A good fat** | 5(1.2) | 3(2.3) | 2(0.7) |  |
| **Cholesterol** | 66 (16.5) | 18(13.8) | 48(17.7) |  |
| **I don't know** | 119 (29.7) | 59(45.4) | 60(22.1) |  |
| **Are trans fats good for health?**  **(no)** | 378 (94.3) | 118(90.8) | 260(95.9) | 0.037** |
| **Reducing trans-fat intake will improve overall health (yes)** | 243 (60.6) | 63(48.5) | 180(66.4) | 0.002** |
| **Do you think listing of trans fat information on food label is mandatory? (yes)** | 327 (81.5) | 98(75.4) | 229(84.5) | 0.028** |
| **Which of these foods, if any, contain trans fats? ^a^** |  |  |  |  |
| **French fries** | 260 (64.8) | 73(56.2) | 187(69) | 0.012** |
| **Lard** | 148 (36.9) | 47(36.2) | 101(37.3) | 0.828 |
| **Butter** | 196 (48.9) | 60(46.2) | 136(50.2) | 0.45 |
| **Non-hydrogenated**  **margarine** | 99 (24.7) | 29(22.3) | 70(25.8) | 0.444 |
| **Hard margarine** | 164 (40.9) | 39(30.0) | 125(46.1) | 0.002** |
| **Vegetable shortening** | 95 (23.7) | 25(19.2) | 70(25.8) | 0.146 |
| **Doughnuts** | 214 (53.4) | 55(42.3) | 159(58.7) | 0.002** |
| **Candies and desserts** | 198 (49.4) | 58(44.6) | 140(51.7) | 0.187 |
| **Pastries/bakery products** | 151(37.7) | 34(26.2) | 117(43.2) | 0.001** |

| **Cookies** | **170 (42.4)** | **45(34.6)** | **125(46.1)** | **0.029**** |
| --- | --- | --- | --- | --- |
| **Crackers** | 136 (33.9) | 39(30.0) | 97(35.8) | 0.251 |
| **Breakfast cereals** | 42 (10.5) | 12(9.2) | 30(11.1) | 0.573 |
| **Poultry/eggs** | 41 (10.2) | 18(13.8) | 23(8.5) | 0.097 |
| **Meat products** | 69 (17.2) | 26(20.0) | 43(15.9) | 0.305 |
| **Seafoods** | 25 (6.2) | 9(6.9) | 16(5.9) | 0.693 |
| **Milk and dairy products** | 54 (13.5) | 20(15.4) | 34(12.5) | 0.436 |
| **Processed meats** | 174 (43.4) | 48(36.9) | 126(46.5) | 0.07 |
| **Processed foods** | 179 (44.60) | 49(37.7) | 130(48) | 0.053 |
| **Dip/salad dressing** | 69 (17.20) | 24(18.5) | 45(16.6) | 0.645 |
| **Trans fat should be limited?** **(yes)** | 293 (73.1) | 83(63.8) | 210(77.5) | 0.011** |
| **Trans fat improves the taste of food** **(yes)** | 196 (48.9) | 59(45.4) | 137(50.6) | <0.001** |
| **Trans fat may increase LDL**  **(yes)** | 304 (75.8) | 84(64.6) | 220(81.2) | 0.001** |
| **Trans fat may increase hypertension (yes)** | 245 (61.1) | 62(47.7) | 183(67.5) | 0.001** |
| **Trans fat may increase diabetes**  **(yes)** | 215 (53.6) | 60(46.2) | 155(57.2) | 0.071 |
| **Trans fat may increase risk of obesity (yes)** | 316 (78.8) | 90(69.2) | 226(83.4) | 0.003** |
| **Trans fat is bad for heart health**  **(yes)** | 305 (76.1) | 91(70.0) | 214(79.0) | 0.11 |

*Chi-Square Test; **significant p-value<0.05; LDL: Low-density lipoprotein. The correct answers are provided in brackets next to each variable. ^a^: Multiple choices were possible, hence total may exceed 100%

Table 3: Behaviors practices related to trans-fat in a sample of Lebanese adults (n=401) by gender

| **Behaviors practices** | **Total *n (%)*** | **Gender** | | ***P-Value* *** |
| --- | --- | --- | --- | --- |
|  |  | Males *n* (%) | Females *n* (%) |  |
| **Which of the following specific types of information that relate to heart health do you look for on food or beverage labels or packages?^a^** |  |  |  |  |
| **Sodium** | 99(24.7) | 20(15.4) | 79(29.2) | 0.003** |
| **Total fats** | 177(44.1) | 56(43.1) | 121(44.6) | 0.767 |
| **Total calories** | 163(40.6) | 47(36.2) | 116(42.8) | 0.204 |
| **Cholesterol** | 198(49.4) | 69(53.1) | 129(47.6) | 0.305 |
| **Trans fats** | 141(35.2) | 41(31.5) | 100(36.9) | 0.293 |
| **Serving size** | 84(20.9) | 18(13.8) | 66(24.4) | 0.016** |
| **Saturated fats** | 141(35.2) | 49(37.7) | 92(33.9) | 0.462 |
| **Statement about heart health** | 103(25.7) | 28(21.5) | 75(27.7) | 0.188 |
| **Partially hydrogenated oils** | 78(19.5) | 17(13.1) | 61(22.5) | 0.026** |
| **Hydrogenated oils** | 88(21.9) | 21(16.2) | 67(24.7) | 0.052 |
| **None of the above** | 62(15.5) | 24(18.5) | 38(14.0) | 0.25 |
| **Are you trying to consume less trans-fat? (yes)** | 291(72.6) | 89(68.5) | 202(74.5) | 0.202 |
| **Would you stop eating your favorite snack food if you knew it had trans-fat in it? (yes)** | 211(52.6) | 60(46.2) | 151(55.7) | 0.073 |
| **How many times a week do you eat fried foods and baked goods such as fries, doughnuts, pastries, cakes, and cookies?** |  |  |  |  |
| **0 times /week.** | 22 (5.5) | 2(1.5) | 20(7.4) | 0.005** |
| **1-2 times / week** | 237 (59.1) | 73(56.2) | 164(60.5) |  |
| **3-4 times/week** | 97 (24.2) | 31(23.8) | 66(24.4) |  |
| **5 times/week** | 24 (6.0) | 13(10.0) | 11(4.1) |  |
| **>5 times/week** | 21 (5.2) | 11(8.5) | 10(3.7) |  |
| **How often do you use canola oil in meal preparation?** |  |  |  |  |
| **Never or Rarely** | 240(59.5) | 73(56.2) | 167(61.6) | 0.195 |
| **Sometimes** | 105(26.2) | 33(25.4) | 72(26.6) |  |
| **Usually or Always** | 56(14.0) | 24(18.5) | 32(11.8) |  |
| **How often do you use palm oil in meal preparation?** |  |  |  |  |
| **Never or Rarely** | 323 (80.5) | 90(69.2) | 233(86.0) | <0.001** |
| **Sometimes** | 64 (16.0) | 31(23.8) | 33(12.2) |  |
| **Usually or Always** | 14(3.5) | 9(6.9) | 5(1.8) |  |

| **How often do you use olive oil in meal preparation?** |  |  |  |  |
| --- | --- | --- | --- | --- |
| **Never or Rarely** | 23(5.7) | 14(10.8) | 9(3.3) | 0.009** |
| **Sometimes** | 118 (29.4) | 39(30.0) | 79(29.2) |  |
| **Usually or Always** | 260 (64.8) | 77(59.2) | 183(67.5) |  |
| **How often do you use corn oil in meal preparation?** |  |  |  |  |
| **Never or Rarely** | 153 (38.2) | 34(26.2) | 119(43.9) | 0.002** |
| **Sometimes** | 145 (36.2) | 59(45.4) | 86(31.7) |  |
| **Usually or Always** | 103(25.7) | 37(28.5) | 66(24.4) |  |
| **How often do you use sunflower oil in meal preparation?** |  |  |  |  |
| **Never or Rarely** | 57(14.2) | 17(13.1) | 40(14.8) | 0.806 |
| **Sometimes** | 146(36.4) | 50(38.5) | 96(35.4) |  |
| **Usually or Always** | 198(49.4) | 63(48.5) | 135(49.8) |  |
| **How often do you use margarine in meal preparation?** |  |  |  |  |
| **Never or Rarely** | 271 (67.6) | 85(65.4) | 186(68.6) | 0.659 |
| **Sometimes** | 105 (26.2) | 35(26.9) | 70(25.8) |  |
| **Usually or Always** | 25(6.2) | 10(7.7) | 15(5.5) |  |
| **How often do you use butter in meal preparation?** |  |  |  |  |
| **Never or Rarely** | 98(24.2) | 35(26.9) | 63(23.2) | 0.651 |
| **Sometimes** | 244 (60.8) | 75(57.7) | 169(62.4) |  |
| **Usually or Always** | 59(14.7) | 20(15.4) | 39(14.4) |  |
| **How often do you use lard/pork in meal preparation?** |  |  |  |  |
| **Never or Rarely** | 341 (85.0) | 101(77.7) | 240(88.6) | 0.004** |
| **Sometimes** | 45 (11.2) | 19(14.6) | 26(9.6) |  |
| **Usually or Always** | 15(3.7) | 10(7.7) | 5(1.8) |  |
| **Have you changed your food purchases to reduce your trans fat intake?(yes)** | 192(47.9) | 55(42.3) | 137(50.6) | 0.122 |
| **Have you purchased foods labelled “0 trans fat”? (yes)** | 169(42.1) | 56(43.1) | 113(41.7) | 0.793 |
| **Have you read Nutrition Facts panels to choose foods with a low trans-fat content? (yes)** | 160(39.3) | 45(34.6) | 115(42.4) | 0.134 |
| **Have you reduced your intake of high fat foods? (yes)** | 277(69.1) | 81(62.3) | 196(72.3) | 0.042** |
| **Have you read ingredients lists to select foods with no partially hydrogenated fats? (yes)** | 151(37.7) | 44(33.8) | 107(39.5) | 0.275 |

| **How often do you ask the server about the type of oils or fat used to prepare a food** |  |  |  |  |
| --- | --- | --- | --- | --- |
| **Never or Rarely** | 321 (80.0) | 96(73.8) | 225(83) | 0.082 |
| **Sometimes** | 68 (17.0) | 28(21.5) | 40(14.8) |  |
| **Usually or Always** | 12(3.0) | 6(4.6) | 6(2.2) |  |
| **How often do you request ingredient or nutrition information of items on the menu** |  |  |  |  |
| **Never or Rarely** | 232(75.9) | 71(54.6) | 161(59.4) | 0.651 |
| **Sometimes** | 145(36.2) | 51(39.2) | 94(34.7) |  |
| **Usually or Always** | 24(6.0) | 8(6.2) | 16(5.9) |  |
| **How often do you order an item on the menu because it was marked as being healthier in some way** |  |  |  |  |
| **Never or Rarely** | 150 (37.4) | 47(36.2) | 103(38) | 0.757 |
| **Sometimes** | 211 (52.6) | 68(52.3) | 143(52.8) |  |
| **Usually or Always** | 17(4.2) | 8(6.2) | 9(3.3) |  |

*Chi-Square Test; **Significant p-value<0.05
The correct answers are provided in brackets next to each variable.
^a^: Multiple choices were possible, hence total may exceed 100%
